# Expression of the MHC class II in triple-negative breast cancer is associated with tumor-infiltrating lymphocytes and interferon signaling

**DOI:** 10.1371/journal.pone.0182786

**Published:** 2017-08-17

**Authors:** In Ah Park, Seong-Hye Hwang, In Hye Song, Sun-Hee Heo, Young-Ae Kim, Won Seon Bang, Hye Seon Park, Miseon Lee, Gyungyub Gong, Hee Jin Lee

**Affiliations:** 1 Departments of Pathology, Asan Medical Center, Seoul, Korea; 2 Asan Center for Cancer Genome Discovery, Asan Institute for Life Sciences, University of Ulsan College of Medicine, Asan Medical Center, Seoul, Korea; University of South Alabama Mitchell Cancer Institute, UNITED STATES

## Abstract

Tumor-infiltrating lymphocytes (TILs) have been known for their strong prognostic and predictive significance in triple-negative breast cancer (TNBC). Several mechanisms for TIL influx in TNBC have been elucidated. Major histocompatibility complex class II (MHC-II) is an essential component of the adaptive immune system and is generally restricted to the surface of antigen-presenting cells. However, it has been reported that interferon-gamma signaling may induce MHC-II in almost all cell types, including those derived from cancer. We aimed to examine the relationship between MHC-II expression in tumor cells and the amount of TILs in 681 patients with TNBC. Further, the prognostic significance of MHC-II and the association of MHC-II with a couple of molecules involved in the interferon signaling pathway were investigated using immunohistochemical staining. Higher MHC-II expression in tumor cells was associated with the absence of lymphovascular invasion (*p* = 0.042); larger amounts of TILs (*p* < 0.001); frequent formations of tertiary lymphoid structures (*p* < 0.001); higher expression of myxovirus resistance gene A, one of the main mediators of the interferon signaling pathway (*p* < 0.001); and higher expression of double-stranded RNA-activated protein kinase, which can be induced by interferons (*p* = 0.008). Moreover, tumors that showed high MHC class I expression and any positivity for MHC-II had larger amounts of CD4- and CD8-positive T lymphocytes (*p* < 0.001). Positive MHC-II expression in tumor cells was associated with better disease-free survival in patients who had lymph node metastasis (*p* = 0.009). In conclusion, MHC-II expression in tumor cells was closely associated with an increase in TIL number and interferon signaling in TNBC. Further studies are warranted to improve our understanding regarding TIL influx, as well as patients’ responses to immunotherapy.

## Introduction

Triple-negative breast cancer (TNBC) is a clinical subtype of invasive tumors that have low or no expression of hormone receptors, such as estrogen receptor (ER) and progesterone receptor (PR), and no overexpression of human epidermal growth factor receptor 2 (HER2). Patients with TNBC, an aggressive type of cancer, are not candidates for ER- or HER2-targeted therapy. Most patients receive adjuvant or neoadjuvant chemotherapy with or without local radiotherapy [1].

In our previous study, we revealed that the expression of major histocompatibility complex (MHC) class I in TNBC is associated with tumor-infiltrating lymphocytes (TILs) and interferon (IFN) signaling in tumor cells [2]. We also reported that one of the main mediators of IFN signaling, myxovirus resistance gene A (MxA), is associated with TILs and is a prognostic factor for TNBC [3]. The presence of TILs can be assessed through histologic examination, immunohistochemistry, and genomic methodologies. The concluding remarks from many TIL studies on breast cancer show that the immune response of patients has a positive effect on therapy response, progression-free survival, and overall survival, especially in TNBC [4–9]. So far, the importance of TILs is usually emphasized by the roles of CD8-positive T cells. However, CD4-positive T cells also have an important role in the antitumor response in both mice and humans. After immunization with cancer cells or specific peptides, tumor suppression depends on a functional CD4-positive T cell effector compartment [10]. Helper T cells have been shown to directly mobilize effector cytotoxic T lymphocytes to virus-infected tissues [11]. Such interaction might be crucial in some cancers.

Antigen presentation by MHC-II, also known as human leukocyte antigen (HLA)-II (HLA-DR, -DP, and -DQ) in humans with co-chaperones HLA-DM and the invariant chain (Ii), is essential for antitumor immunity and the development of adaptive immune responses [12–15]. Generally, MHC-II expression is known to be restricted to professional antigen presenting cells, such as dendritic cells, macrophages, and B cells, but is inducible through IFN-γ signaling in almost all cell types, including those derived from cancer [16, 17]. Recently, the capability of CD4-positive T cells to directly kill MHC-II-expressing target cells in an MHC-II–CD4-restricted manner, independent of B, NK, or other T cells in the host, has been reported [18–20].

Although HLA-II expression in breast cancer has been previously reported, most of the studies have analyzed only a small number of breast cancer tissues or conducted whole transcriptome analysis using RNA sequencing, which might have represented HLA-II expression in immune cells rather than cancer cells [21–24]. In the current study, we evaluated the expression of MHC-I, MHC-II (HLA-DR, -DP, and -DQ), and IFN-associated molecules in tumor cells; TIL percentage; and the number of CD4- and CD8-expressing cells in a large number of TNBC cases. This study elucidates the role of HLA-II expression in TNBC tumor cells.

## Materials and methods

### Patients and tissue specimens

A total of 681 preoperative chemo- and radiotherapy naive-patients with TNBC who underwent surgery for primary breast cancer from 2004 to 2010 at the Asan Medical Center, Seoul, Korea, were included in this study. All patients underwent adjuvant systemic treatment, while 550 patients (80.8%) were treated with adjuvant radiotherapy. Of the 681 patients, 471 (69.2%) were treated with four cycles of adjuvant anthracycline and cyclophosphamide (AC) (adriamycin 60 mg/m^2^ and cyclophosphamide 600 mg/m^2^). The remaining 210 patients were treated with four cycles of AC followed by either four cycles of paclitaxel (175 mg/m^2^) or four cycles of docetaxel (75 mg/m^2^). Those who had lymph node metastasis were treated with AC and taxane, whereas those who did not were treated with AC. We obtained the patients’ clinicopathological information from their medical records and surgical pathology reports. The Institutional Review Board of Asan Medical Center approved this study for the exemption from informed consent after de-identification of information.

The definition of TNBC in terms of the expression of biomarkers, including ER, PR, and HER2, and *HER2* gene amplification recognized by fluorescence *in situ* hybridization or silver *in situ* hybridization was the same as previously described [25].

### Histological evaluation

Hematoxylin-and-eosin (H&E)-stained slides were reviewed by two pathologists (H.J.L. and G.G.). The slides were then histopathologically analyzed for TILs (defined as the percentage of stroma of invasive carcinoma infiltrated by lymphocytes in 10% increments; if less than 10% of the stroma was infiltrated by TILs, 1% or 5% was used; all available full sections were evaluated) [26], the amount of inflammatory cells at the border of the tumor’s invasive area (according to Klintrup criteria; score 0, no inflammatory cells at the invasive border; score 1, mild and patchy increase of inflammatory cells; score 2, increased inflammatory reaction forming a band-like infiltrate at the invasive border; score 3, prominent inflammatory cells forming a cup-like zone at the invasive border) [27], the amount of tertiary lymphoid structures (TLSs) in the adjacent area of the invasive and *in situ* components of the tumor (none, no TLS formation in the area adjacent to the tumor; little, TLSs occupying an area of less than 10% of the circumference of the tumor; moderate, 10% to 50%; abundant, over 50%) [25], histologic subtype and grade, tumor size, pathological tumor stage (pT), pathological lymph node stage (pN), and lymphovascular invasion. TLSs were confirmed when lymphoid aggregates showed vessels having high endothelial venule-like features (plump and cuboidal endothelium) with or without germinal centers. Histological type was defined based on the 2012 WHO classification criteria, while histological grade was assessed using the modified Bloom–Richardson classification [28].

### Clinicopathological characteristics of the study population

The median age of the patients at the time of diagnosis was 47.4 years (range, 23 to 76 years). Tumor sizes of invasive portion ranged from 0.3 to 9 cm (median, 2.5 cm). There were 299 cases of pT1, 356 cases of pT2, 25 cases of pT3, and 1 case of pT4. In terms of pathological lymph node stage (pN), 472, 121, 46, and 42 cases were pN0, pN1, pN2, and pN3, respectively.

### Tissue microarray construction and immunohistochemical evaluation

**Fig 1:**
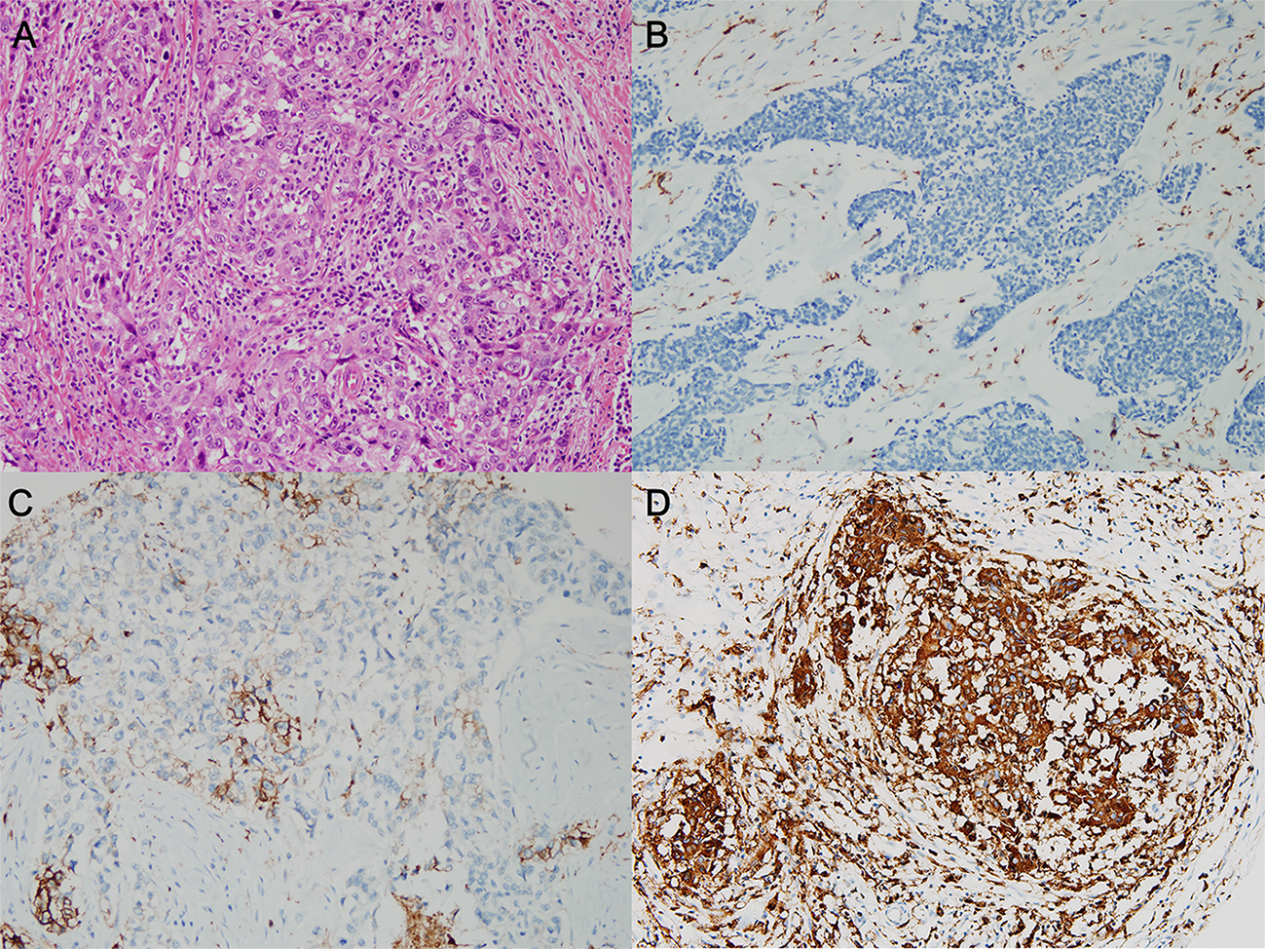
**Representative image of triple-negative breast cancer** (A) hematoxylin-and-eosin-stained slide and the three degrees of major histocompatibility complex (MHC) class II expression in tumor cells. (B) No MHC-II expression and (C) lower and (D) higher expression than the mean value of the immunoreactive score, which was generated as the product of intensity and percentage of positively stained cells.

Formalin-fixed, paraffin-embedded tissue samples were arrayed using a tissue-arraying instrument, as previously described [29]. Each sample was arrayed in three 1-mm-diameter cores to minimize tissue loss and overcome tumor heterogeneity. Tissue microarray sections were stained using an automatic immunohistochemical staining device (Benchmark XT; Ventana Medical Systems, Tucson, AZ). Antibodies for MHC-I (HLA-ABC; 1:1600; ab70328; EMR8-5; Abcam, Cambridge, UK), MHC-II (HLA-DR, -DP, and -DQ; 1:1000; CR3/43; Santa Cruz Biotechnology, Santa Cruz, CA), MxA (1:1000; ab95926; Abcam), double-stranded RNA-activated protein kinase (PKR, 1:400; ab32052; Abcam), CD4 (1:4; Ventana Medical Systems), and CD8 (1.200; DAKO, Glostrup, Denmark) were used. All protein expression except CD4 and CD8 was evaluated using a four-value intensity score (0, 1, 2, and 3) in tumor cells. The percentage of membranous and/or cytoplasmic expression was also evaluated. The “immunoreactive score” was derived from the product of intensity and positively stained tumor cell percentage. Immunoreactive scores were dichotomized by the mean value of the expression of each protein, while tumors having immunoreactive scores of zero were designated as “no” expression (Fig 1).

The immunostained tissue microarray slides for CD4 and CD8 were scanned using a digital microscope scanner (Pannoramic 250 FLASH, 3DHISTECH Ltd., Budapest, Hungary). The number of CD4- and CD8-positive cells in tissue microarray cores was determined using the NuclearQuant module of the Pannoramic Viewer 1.15.2 (3DHISTECH Ltd). Representative images of immunohistochemical staining for CD4 and CD8 are shown in S1 Fig.

### Statistical analysis

All statistical analyses were performed using SPSS statistical software (version 18; SPSS, Chicago, IL). The Kruskal–Wallis test, Mann–Whitney test, linear-by-linear association test, log-rank test, and Cox proportional hazards regression model were used as appropriate. All tests were two-sided, and statistical significance was set at 5%.

## Results

### Higher MHC-II expression in tumor cells is associated with larger amounts of tumoral and peritumoral lymphocytes, and molecules involved in interferon signaling pathway

The expression level of MHC-II in tumor cells was dichotomized by its mean value using immunohistochemical staining. We divided the tumors into three groups, those with no (479 cases, 70.3%), low (86 cases, 12.6%), and high (116 cases, 17.0%) expression of MHC-II molecules, to compare the clinicopathological variables according to the expression level (Table 1). Tumors with higher MHC-II expression were shown to have lower lymphovascular invasion (*p* = 0.042), larger amounts of TILs (*p* < 0.001), higher Klintrup criteria scores (*p* < 0.001), and larger amounts of TLSs adjacent to the invasive tumor area (*p* < 0.001). Programmed death-ligand 1 (PD-L1) expression in any tumor cell was significantly associated with higher expression levels of MHC-II (*p* < 0.001). Higher expression of MxA, one of the main mediators of the IFN signaling pathway, was correlated with higher MHC-II expression (*p* < 0.001). Expression of PKR, which has numerous physiological and pathological functions during cellular stress and can be induced by IFN signaling, also showed a positive correlation with MHC-II expression (*p* = 0.008). These results suggest that MHC-II expression in tumor cells is associated with not only tumoral and peritumoral lymphocytic infiltration but also the IFN signaling pathway.

**Table 1:** Comparison of clinicopathological variables according to MHC-II expression in tumor cells.

	MHC-II expression			
	No (n = 479)	Low* (n = 86)	High* (n = 116)	*p* value
Histological grade				**0.041**
1	0	0	0 (0.0%)	
2	21 (18.1%)	14 (16.3%)	126 (26.3%)	
3	95 (81.9%)	72 (83.7%)	353 (73.7%)	
Pathologic T stage				0.074
1	213 (44.5%)	45 (52.3%)	41 (35.3%)	
2	243 (50.7%)	40 (46.5%)	73 (62.9%)	
3	22 (4.6%)	1 (1.2%)	2 (1.7%)	
4	1 (0.2%)	0	0	
Lymph node metastasis				0.726
Negative	332 (69.3%)	57 (66.3%)	83 (71.6%)	
Positive	147 (30.7%)	29 (33.7%)	33 (28.4%)	
Lymphovascular invasion				**0.042**
Negative	355 (74.1%)	69 (80.2%)	98 (84.5%)	
Positive	124 (25.9%)	17 (19.8%)	18 (15.5%)	
TILs (%)				**<0.001**
≤ 10	144 (30.1%)	13 (15.1%)	8 (6.9%)	
20–30	118 (24.6%)	16 (18.6%)	20 (17.2%)	
40–60	95 (19.8%)	19 (22.1%)	34 (29.3%)	
>60	122 (25.5%)	38 (44.2%)	54 (46.6%)	
Peritumoral lymphocytes (Klintrup criteria)				**<0.001**
0	40 (8.4%)	2 (2.3%)	0 (0.0%)	
1	194 (40.5%)	21 (24.4%)	21 (18.1%)	
2	184 (38.4%)	45 (52.3%)	57 (49.1%)	
3	61 (12.7%)	18 (20.9%)	38 (32.8%)	
TLS adjacent to the invasive area				**<0.001**
No	50 (10.4%)	4 (4.7%)	1 (0.9%)	
Mild	85 (17.7%)	12 (14.1%)	12 (10.3%)	
Moderate	173 (36.1%)	36 (41.9%)	42 (36.2%)	
Severe	171 (35.7%)	34 (39.5%)	61 (52.6%)	
MHC class I expression				**<0.001**
Low	271 (58.0%)	33 (38.4%)	11 (9.5%)	
High	196 (42.0%)	53 (61.6%)	105 (90.5%)	
PD-L1 expression				**<0.001**
Negative	440 (96.7%)	74 (87.1%)	83 (71.6%)	
Positive (any)	15 (3.3%)	11 (12.9%)	33 (28.4%)	
MxA expression				**<0.001**
Low	321 (67.9%)	48 (55.8%)	50 (43.1%)	
High	152 (32.1%)	38 (44.2%)	66 (56.9%)	
PKR expression				**0.008**
Low	256 (54.6%)	37 (43.0%)	47 (40.5%)	
High	213 (45.4%)	49 (57.0%)	69 (59.5%)	

*The expression of MHC-II molecules was dichotomized into low and high groups using the mean of the immunoreactive score, 27.2; MxA, myxovirus resistance A; PKR, double-stranded RNA-activated protein kinase; TILs, tumor infiltrating lymphocytes; TLS, tertiary lymphoid structure.

In addition, we analyzed the association between clinicopathological variables and MHC-II expression in tumor cells after dividing the patients into two groups according to lymph node metastasis (S1 Table). Same tendency was observed in both groups.

### Tumors showing high MHC-I expression and positivity for MHC-II have larger amounts of lymphocytes

**Fig 2:**
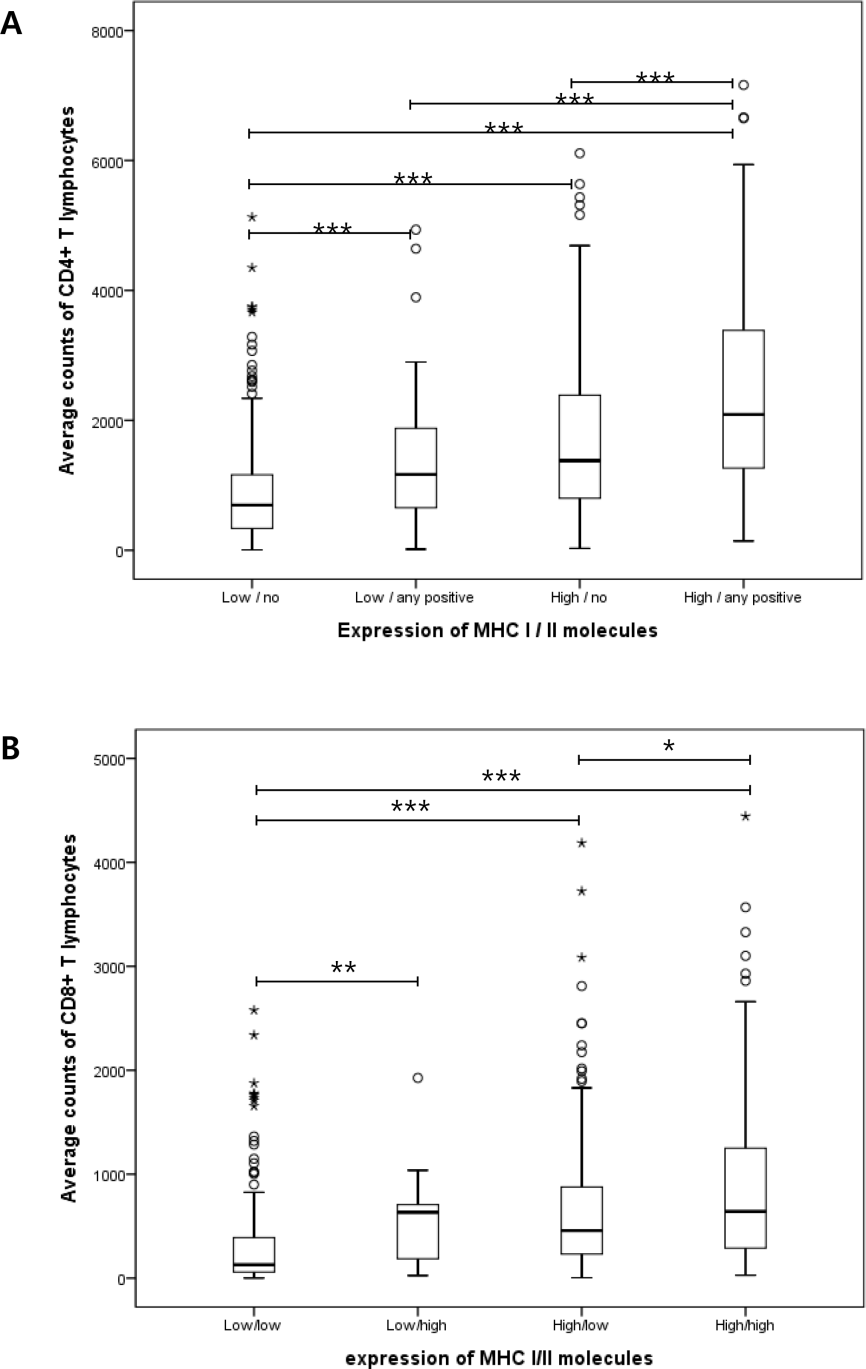
**The amount of CD4-positive (A) and CD8-positive (B) lymphocytes according to protein expression of MHC-I and -II in tumor cells.** The patients were divided into four groups by combining MHC-I and -II expression levels. MHC-I was dichotomized by the mean value of its expression. In case of MHC-II, ‘no’ designates the cases with total negativity for MHC-II, and the others who have tumors with positivity for MHC-II regardless of percentage and intensity are designated as ‘any positive’. (*p < 0.05; **p < 0.01; ***p < 0.001).

Antigen presentation by MHC-I and -II in tumor cells is critical for effective antitumor activity of TILs. We divided the patients into four groups by combining MHC-I and -II expression levels. MHC-I well known to be expressed on the all types of nucleated cells was dichotomized by the mean value of its expression. Whereas, in terms of MHC-II which is normally found only on professional antigen-presenting cells, we divided patients into two groups, one of which included patients who showed total negativity for MHC-II (“no” in Fig 2) and the other of which included patients who had tumors with positivity for MHC-II regardless of percentage and intensity (“any positive” in Fig 2). A total of 271 patients (39.7%) had an MHC-I expression level that was lower than the mean value and no MHC-II expression, while 44 patients (6.4%) showed lower MHC-I expression and positivity for MHC-II in tumor cells. Moreover, 196 (28.7%) patients showed higher MHC-I expression and no MHC-II expression, whereas 158 patients (23.1%) had higher MHC-I expression and positivity for MHC-II. Average amounts of CD4- and CD8-positive lymphocytes were evaluated and compared among these four groups using the Kruskal–Wallis test. We also executed all six possible comparisons for each subset of lymphocytes as post analyses using the Mann–Whitney test. As shown in Fig 2, tumors that showed high MHC-I expression and positivity for MHC-II had significantly larger amounts of CD4- and CD8-positive lymphocytes than all the other groups. However, tumors with either high MHC-I expression or positivity for MHC-II revealed to have more lymphocytes than those with low MHC-I expression and no MHC-II expression.

### Tumor cells with MHC-II expression are associated with better prognosis in patients with lymph node metastasis

Next, we conducted survival analyses using clinicopathological factors that could affect the patients’ clinical outcomes as variables (Table 2). Higher pT, presence of lymph node metastasis and lymphovascular invasion, fewer amounts of TILs, lesser formation of TLSs adjacent to invasive area, and lower expression of MxA were significantly negatively associated with disease-free survival (all *p* ≤ 0.002). Subgroup analysis showed that these negative prognostic factors, except MxA expression, remained statistically significant for patients with lymph node metastasis, whereas pT, lymphovascular invasion, and MxA expression were effective prognostic factors in patients without lymph node metastasis. Expression levels of MHC-I, PD-L1, and PKR in tumor cells did not show a significant association with clinical outcomes.

**Table 2:** Univariate analyses of clinicopathological variables affecting disease-free survival.

Variables	Total (n = 681)			*Lymph node positive (n = 209)			*Lymph node negative (n = 472)		
	HR	95% CI	*p* value	HR	95% CI	*p*value	HR	95% CI	*p* value
Histological grade: 3 vs. 1/2	0.823	0.541–1.251	0.360	1.094	0.568–2.110	0.788	0.583	0.332–1.022	0.059
Pathological T stage: 3/4 vs. 1/2	3.591	1.926–6.695	<0.001	2.129	1.009–4.492	0.047	4.406	1.374–14.131	0.013
Lymph node metastasis: positive vs. negative	2.709	1.867–3.933	<0.001						
Lymphovascular invasion: positive vs. negative	3.341	2.302–4.851	<0.001	2.703	1.535–4.760	0.001	2.343	1.231–4.458	0.010
TILs: 10% increments	0.982	0.974–0.989	<0.001	0.964	0.964–0.987	<0.001	0.985	0.974–0.995	0.005
TLSs adjacent to invasive area: moderate to severe vs. no or mild	0.492	0.336–0.720	<0.001	0.354	0.210–0.594	<0.001	0.678	0.381–1.208	0.184
MHC-I expression: high vs. low or no	0.917	0.631–1.333	0.651	0.716	0.425–1.206	0.210	1.170	0.679–2.014	0.572
MHC-II expression: positive vs. negative	0.713	0.460–1.107	0.130	0.400	0.196–0.814	0.012	1.131	0.635–2.013	0.677
PD-L1 expression: positive vs. negative	0.487	0.198–1.195	0.116	0.620	0.194–1.984	0.416	0.394	0.096–1.620	0.197
MxA expression: high vs. low	0.505	0.327–0.779	0.002	0.577	0.316–1.054	0.074	0.479	0.256–0.896	0.021
PKR expression: high vs. low	0.836	0.573–1.219	0.352	0.793	0.468–1.342	0.387	0.862	0.502–1.480	0.590

CI, confidence interval; HR, hazard ratio; MHC, major histocompatibility complex; MxA, myxovirus resistance gene A; PKR, double-stranded RNA-activated protein kinase; TILs, tumor-infiltrating lymphocytes; TLSs, tertiary lymphoid structures.

*In the columns, lymph node positive and negative refer to patients who have and do not have lymph node metastasis, respectively.

**Fig 3:**
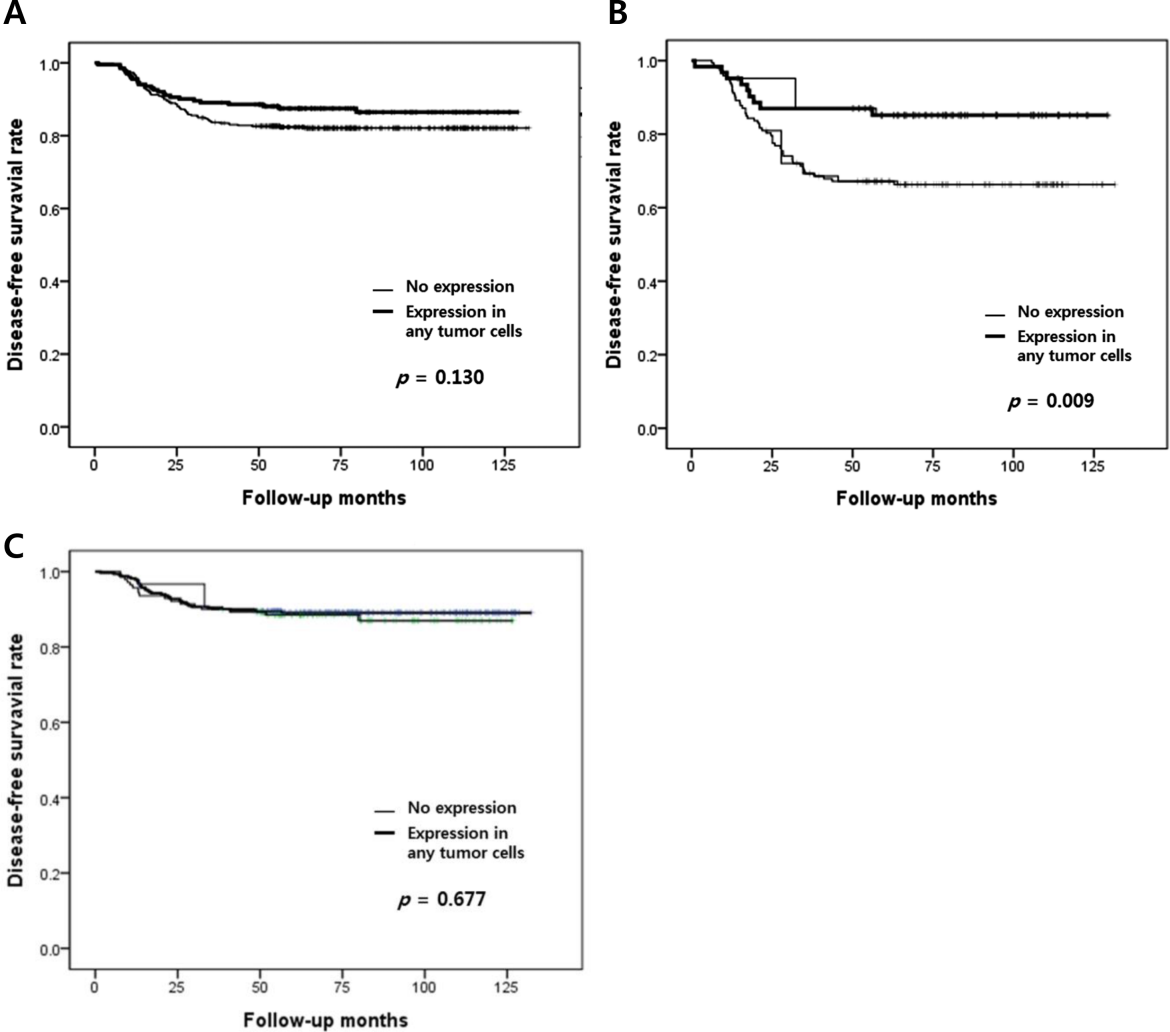
Kaplan–Meier survival curves according to MHC-II expression in tumor cells. (A) All patients, (B) patients with lymph node metastasis, and (C) patients without lymph node metastasis.

As shown in Fig 3, patients having tumors with any positive expression of MHC-II tended to have better survival rates, though the relationship was not statistically significant (*p* = 0.130). When we compared Kaplan-Meyer survival curves of the four subgroups in combination of MHC-I (low/high) and MHC-II (no/positive), tumors with no MHC-II expression tended to have worse prognosis regardless of MHC-I status (data not shown), although this result was also not statistically significant. However, in patients who had lymph node metastasis, any MHC-II expression in tumor cells was significantly associated with better disease-free survival (*p* = 0.009). After multivariate analysis including MHC-II expression, pT stage, lymphovascular invasion, level of TILs, and TLSs, MHC-II expression was not an independent prognostic factor. Remnant variables which have been found to be independent prognostic factors in total patients as well as lymph node-positive and -negative subgroups are described in S2 Table.

## Discussion

The present study showed that 29.7% of TNBC cells expressed MHC-II. A previous study that performed immunohistochemical analysis with anti-CD74 (HLA class II histocompatibility antigen gamma chain) and anti-HLA-DPB1 in TNBC tumor specimens [22] revealed that all five examined specimens had CD74 protein expression in tumor cells. The staining was cytoplasmic, membranous, or both, with 5% to 90% of tumor cells showing immune reactivity that was weak to moderate in intensity. HLA-DPB1 protein expression was noted in two of five TNBC tumors, with weak-to-moderate cytoplasmic and membranous staining in 20% and 40% of tumor cells, respectively. The expression of HLA-DR and HLA-DQ from breast cancer biopsies was also analyzed in 52 invasive ductal carcinomas using immunohistochemistry [23]. Moreover, 86.5% of cancers showed high levels of HLA-DQ, whereas 69.2% showed low levels of HLA-DR expression. In another study, HLA-DR expression was examined using immunohistochemical methods in five medullary breast carcinomas, six atypical medullary carcinomas, and 20 invasive ductal carcinomas [24]. Expression was most dominant in medullary carcinomas, followed by atypical medullary carcinomas and invasive duct carcinomas with and without lymphocytic infiltrates. The mean intensity and percentage of HLA-DR tumor immunostaining were significantly higher in medullary carcinomas than in the other three tumor groups. These results are concordant with those of the present study, given the significant correlation between MHC-II expression in tumor cells and the level of TILs in TNBCs.

We found prognostic significance of MHC-II expression in TNBCs with lymph node metastasis, although it was not an independent prognostic factor. These results may be explained by the significant correlation between MHC-II expression in tumor cells and TIL level, which is a strong prognostic factor for patients with TNBC. However, prognostic significance of MHC-II expression has also been shown in patients with melanoma receiving anti-programmed cell death protein 1 (PD-1) therapy and those with diffuse large B-cell lymphoma receiving rituximab, cyclophosphamide, doxorubicin, vincristine, and prednisone [30, 31]. These findings suggest that increased MHC-II expression (and in turn increased antigen presentation) in tumor cells facilitates better antitumor response in the tumor microenvironment.

Current evidence has suggested that CD8-positive cytotoxic lymphocytes rely on CD4-positive T cells and can moderate the destruction of tumor cells [32–36]. Therefore, antigenic epitope presentation of both MHC-I and -II (MHC-I/II) induces high affinity T cells that respond to the MHC-I/II epitopes [37]. It has also been reported that naive tumor-specific CD4-positive T cells differentiate in vivo and eradicate established melanoma, tumor-responsive CD4-positive T cells improve cytotoxic activity and obliterate large established melanoma after transferring into lymphopenic hosts, and MHC-II-restricted cytotoxic T lymphocytes are generated by mature CD4-positive T helper cells [18–20]. Recently, effectiveness of neoantigen-derived cancer vaccines in two studies with melanoma patients has been described [38, 39]. Both studies showed dominant CD4-positive T cell responses to neo-epitopes. Therefore, MHC-II expression in tumor cells and its close association with CD4-positive T cells in our study suggests the possible cytotoxic activity of CD4-positive T cells on MHC-II expression in TNBC cells.

In the present study, we found a significant positive correlation between PD-L1 and MHC-II expression in TNBC. Recently, the safety and antitumor activity of the PD-1 inhibitor was assessed in a phase Ib trial involving patients with advanced TNBC [40]. Johnson et al. showed that HLA-DR positivity in tumor cells is related to a therapeutic response, as well as the amount of tumor infiltrated CD4- and CD8-positive T cells in anti-PD-1-treated patients with melanoma [41]. They also found that MHC-II-positive melanoma cell lines demonstrate signatures of “PD-1 signaling,” “allograft rejection,” and “T-cell receptor signaling,” among others using pathway analysis. Therefore, they suggested that MHC-II-positive tumors can be identified through immunohistochemistry using commercially available antibodies for MHC-II to improve anti-PD-1 patient selection.

A limitation of our study includes the heterogeneous expression of MHC-II. Given our use of tissue microarray to assess MHC-II expression in tumor cells, we cannot exclude the possibility that MHC-II-expressing tumor cells exist in other parts of the cancer tissue. Further studies exploring the role of MHC-II expression in tumor cells are warranted to discover effective ways of improving patients’ immune responses to cancer.

## Supporting information

S1 Fig **Representative images of immunohistochemical staining for (A&B) CD4 and (C&D) CD8.** The numbers of CD4- and CD8-positive cells in tissue microarray cores were substantially different among the cases, which were determined using half-automatic program.
(PDF)

S1 Table Comparison of clinicopathological variables according to MHC-II expression in tumor cells and lymph node metastasis. (DOCX)

S2 Table Multivariate analyses of clinicopathological variables affecting disease-free survival. (DOCX)
