# Supplementary figures and images for: Expression of the MHC class II in triple-negative breast cancer is associated with tumor-infiltrating lymphocytes and interferon signaling
Source: PLoS One. 2017 Aug 17;12(8):e0182786. doi: 10.1371/journal.pone.0182786 (PMC5560630; doi:10.1371/journal.pone.0182786)

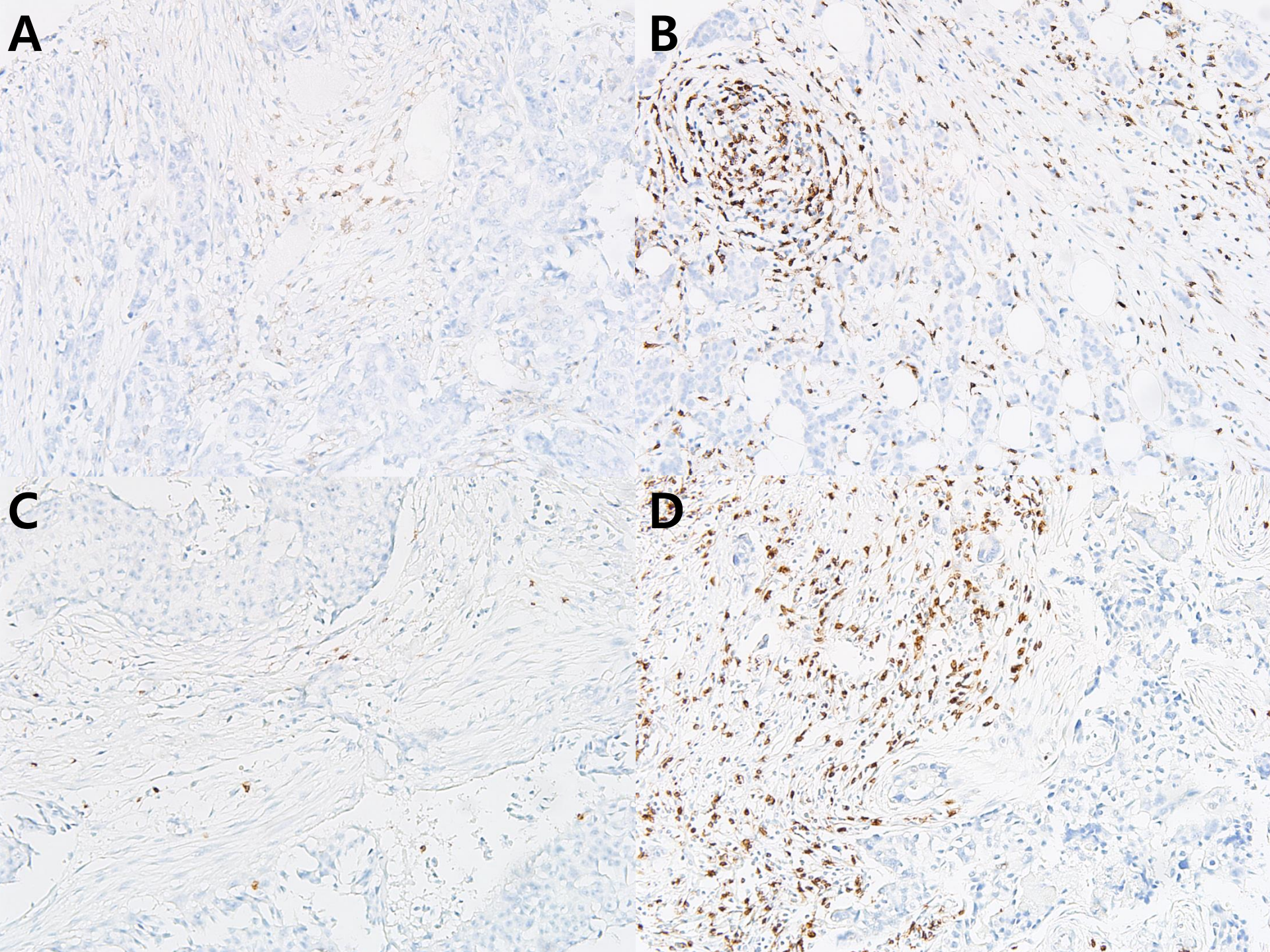

Supplement: S1 Fig — Representative images of immunohistochemical staining for (A&B) CD4 and (C&D) CD8. The numbers of CD4- and CD8-positive cells in tissue microarray cores were substantially different among the cases, which were determined using half-automatic program. (PDF) [file pone.0182786.s001.pdf]
