# Supplementary material for: Expression of the MHC class II in triple-negative breast cancer is associated with tumor-infiltrating lymphocytes and interferon signaling
Source: PLoS One. 2017 Aug 17;12(8):e0182786. doi: 10.1371/journal.pone.0182786 (PMC5560630; doi:10.1371/journal.pone.0182786)
Supplement: S1 Table — (DOCX) [file pone.0182786.s002.docx]

**S1 Table.** **Comparison of clinicopathological variables according to MHC-II expression in tumor cells and lymph node metastasis.**

|  | Lymph node positive (n=209) | | | Lymph node negative (n=472) | | |
| --- | --- | --- | --- | --- | --- | --- |
|  | MHC-II expression | | *p* value | MHC-II expression | | *p* value |
|  | No  (n = 147) | Any positive (n = 62) |  | No  (n = 332) | Any positive (n = 140) |  |
| Histological grade |  |  | 0.706 |  |  | **0.010** |
| 1 | 0 (0.0%) | 0 (0.0%) |  | 0 (0.0%) | 0 (0.0%) |  |
| 2 | 31 (21.1%) | 11 (17.7%) |  | 95 (28.6%) | 24 (17.1%) |  |
| 3 | 116 (78.9%) | 51 (82.3%) |  | 237 (71.4%) | 116 (82.9%) |  |
| Pathologic T stage |  |  | 0.296 |  |  | 0.777 |
| 1 | 39 (26.5%) | 16 (25.8%) |  | 174 (52.4%) | 70 (50.0%) |  |
| 2 | 91 (61.9%) | 45 (72.6%) |  | 152 (45.8%) | 68 (48.6%) |  |
| 3 | 16 (10.9%) | 1 (1.6%) |  | 6 (1.8%) | 2 (1.4%) |  |
| 4 | 1 (0.7%) | 0 (0.0%) |  | 0 (0.0%) | 0 (0.0%) |  |
| Lymphovascular invasion |  |  | **0.035** |  |  | 0.117 |
| Negative | 66 (44.9%) | 38 (61.3%) |  | 289 (87.0%) | 129 (92.1%) |  |
| Positive | 81 (55.1%) | 24 (38.7%) |  | 43 (13.0%) | 11 (7.9%) |  |
| TILs (%) |  |  | **<0.001** |  |  | **<0.001** |
| ≤ 10 | 37(25.2%) | 3 (4.8%) |  | 107 (32.2%) | 18 (12.9%) |  |
| 20–30 | 42 (28.6%) | 13 (21.0%) |  | 76 (22.9%) | 23 (16.4%) |  |
| 40–60 | 35 (23.8%) | 12 (19.4%) |  | 60 (18.1%) | 41 (29.3%) |  |
| >60 | 33 (22.4%) | 34 (54.8%) |  | 89 (26.8%) | 58 (41.4%) |  |
| Peritumoral lymphocytes (Klintrup criteria) |  |  | **<0.001** |  |  | **<0.001** |
| 0 | 9 (6.1%) | 0 (0.0%) |  | 31 (9.3%) | 2 (1.4%) |  |
| 1 | 67 (45.6%) | 11 (17.7%) |  | 127 (38.3%) | 31 (22.1%) |  |
| 2 | 52 (35.4%) | 28 (45.2%) |  | 132 (39.8%) | 74 (52.9%) |  |
| 3 | 19 (12.9%) | 23 (37.1%) |  | 42 (12.7%) | 33 (23.6%) |  |
| TLS adjacent to the invasive area |  |  | **0.002** |  |  | **0.003** |
| No | 16 (10.9%) | 1 (1.6%) |  | 34 (10.2%) | 4 (2.9%) |  |
| Mild | 27 (18.4%) | 7 (11.3%) |  | 58 (17.5%) | 17 (12.1%) |  |
| Moderate | 47 (32.0%) | 18 (29.0%) |  | 126 (38.0%) | 60 (42.9%) |  |
| Severe | 57 (38.8%) | 36 (58.1%) |  | 114 (34.3%) | 59 (42.1%) |  |
| MHC class I expression |  |  | **<0.001** |  |  | **<0.001** |
| Low | 86 (60.1%) | 12 (19.4%) |  | 185 (57.1%) | 32 (22.9%) |  |
| High | 57 (39.9%) | 50 (80.6%) |  | 139 (42.9%) | 108 (77.1%) |  |
| PD-L1 expression |  |  | **<0.001** |  |  | **<0.001** |
| Negative | 137 (97.2%) | 49 (79.0%) |  | 303 (96.5%) | 108 (77.7%) |  |
| Positive (any) | 4 (2.8%) | 13 (21.0%) |  | 11 (3.5%) | 31 (22.3%) |  |
| MxA expression |  |  | **<0.001** |  |  | **0.001** |
| Low | 107 (73.8%) | 29 (46.8%) |  | 214 (65.2%) | 69 (49.3%) |  |
| High | 38 (26.2%) | 33 (53.2%) |  | 114 (34.8%) | 71 (50.7%) |  |
| PKR expression |  |  | 0.068 |  |  | **0.020** |
| Low | 79 (55.2%) | 25 (40.3%) |  | 177 (54.3%) | 59 (42.1%) |  |
| High | 64 (44.8%) | 37 (59.7%) |  | 149 (45.7%) | 81 (57.9%) |  |

*The expression of MHC-II molecules was dichotomized into low and high groups using the mean of the immunoreactive score, 27.2; MxA, myxovirus resistance A; PKR, double-stranded RNA-activated protein kinase; TILs, tumor infiltrating lymphocytes; TLS, tertiary lymphoid structure.
