# Supplementary material for: Expression of the MHC class II in triple-negative breast cancer is associated with tumor-infiltrating lymphocytes and interferon signaling
Source: PLoS One. 2017 Aug 17;12(8):e0182786. doi: 10.1371/journal.pone.0182786 (PMC5560630; doi:10.1371/journal.pone.0182786)
Supplement: S2 Table — (DOCX) [file pone.0182786.s003.docx]

S2 Table. Multivariate analyses of clinicopathological variables affecting disease-free survival

| Variables | Total (n = 681) | | | *Lymph node positive  (n = 209) | | | *Lymph node negative (n = 472) | | |
| --- | --- | --- | --- | --- | --- | --- | --- | --- | --- |
|  | HR | 95%  CI | *p*  value | HR | 95%  CI | *p*  value | HR | 95% CI | *p* value |
| Pathological T stage: 3/4 vs. 1/2 |  |  |  |  |  |  | 4.105 | 1.264–13.328 | **0.019** |
| Lymph node metastasis: positive vs. negative | 1.959 | 1.279–2.873 | **0.005** |  |  |  |  |  |  |
| Lymphovascular invasion: positive vs. negative | 2.295 | 1.499–3.516 | **<0.001** | 2.249 | 1.271–3.980 | **0.005** | 2.079 | 1.088–3.972 | **0.027** |
| TILs: 10% increments | 0.982 | 0.974–0.990 | **<0.001** | 0.983 | 0.970–0.996 | **0.011** | 0.985 | 0.974–0.996 | **0.006** |
| TLSs adjacent to invasive area: moderate to severe vs. no or mild |  |  |  | 0.597 | 0.327–1.090 | 0.093 |  |  |  |

CI, confidence interval; HR, hazard ratio; TILs, tumor-infiltrating lymphocytes; TLSs, tertiary lymphoid structures.

*In the columns, lymph node positive and negative refer to patients who have and do not have lymph node metastasis, respectively.
